# Chemical Elicitors of Antibiotic Biosynthesis in Actinomycetes

**DOI:** 10.3390/microorganisms6020052

**Published:** 2018-06-08

**Authors:** Anton P. Tyurin, Vera A. Alferova, Vladimir A. Korshun

**Affiliations:** 1 Gause Institute of New Antibiotics, Bolshaya Pirogovskaya 11, 119021 Moscow, Russia; alferovava@gmail.com (V.A.A.); v-korshun@yandex.ru (V.A.K.); 2 Shemyakin-Ovchinnikov Institute of Bioorganic Chemistry, Miklukho-Maklaya 16/10, 117997 Moscow, Russia

**Keywords:** actinomycetes, antibiotic biosynthesis, silent biosynthetic pathways, γ-butyrolactones, HiTES, translation inhibitors

## Abstract

Whole genome sequencing of actinomycetes has uncovered a new immense realm of microbial chemistry and biology. Most biosynthetic gene clusters present in genomes were found to remain “silent” under standard cultivation conditions. Some small molecules—chemical elicitors—can be used to induce the biosynthesis of antibiotics in actinobacteria and to expand the chemical diversity of secondary metabolites. Here, we outline a brief account of the basic principles of the search for regulators of this type and their application.

## 1. Introduction

Since the discovery of streptomycin by Selman A. Waksman, actinomycetes have become one of the most fruitful sources of new antibiotics. Most antibiotic classes in current clinical use were discovered during the “golden era”, 1940–1960s, by phenotypic screening of soil microorganisms. Moreover, since the 1960s, a significant number of approved drugs has been designed using chemical modifications of natural scaffolds. Due to an “innovation gap” in this area, society is now facing an emerging threat of microbial drug resistance. An urgent need for new effective antimicrobials has become an important social and political issue [1,2].

On the other hand, achievements in genome sequencing of actinomycetes has revealed a difference between their potential and observed biosynthetic gene expression. Biosynthetic gene clusters (BGCs) are several generally contiguous genes encoding enzymes responsible for a stepwise assembly of complex bioactive molecules. According to data from the majority of published genomes, most BGCs remain “silent” under standard cultivation conditions. These silent, or cryptic, BGCs represent a potential source of new scaffolds for the discovery of novel antimicrobials [3,4,5]. Several techniques for activation of silent BGCs have been developed in recent decades, e.g., direct identification of BGCs and their expression in heterologous hosts [6,7,8,9,10], and systematic alteration of cultivation parameters (the “one strain—many compounds” (OSMAC) approach) [11,12,13]. These strategies are extremely powerful although still remaining laborious and resource-intensive, especially for large (>40 kb) BGCs. Further techniques comprise co-cultivation [14,15], ribosome engineering [16,17,18], and the use of chemical elicitors—compounds that induce the synthesis of antibiotics in actinomycetes [14,19,20,21]. The last approach is accounted and discussed succinctly in this review, covering literature up to December 2017. Here, we focus on small organic molecules capable in nanomolar to micromolar minimum effective concentrations to induce biosynthesis of secondary metabolite in actinomycetes.

## 2. γ-Butyrolactones (GBL) and Related (auto)Regulators

**Chart 1:**
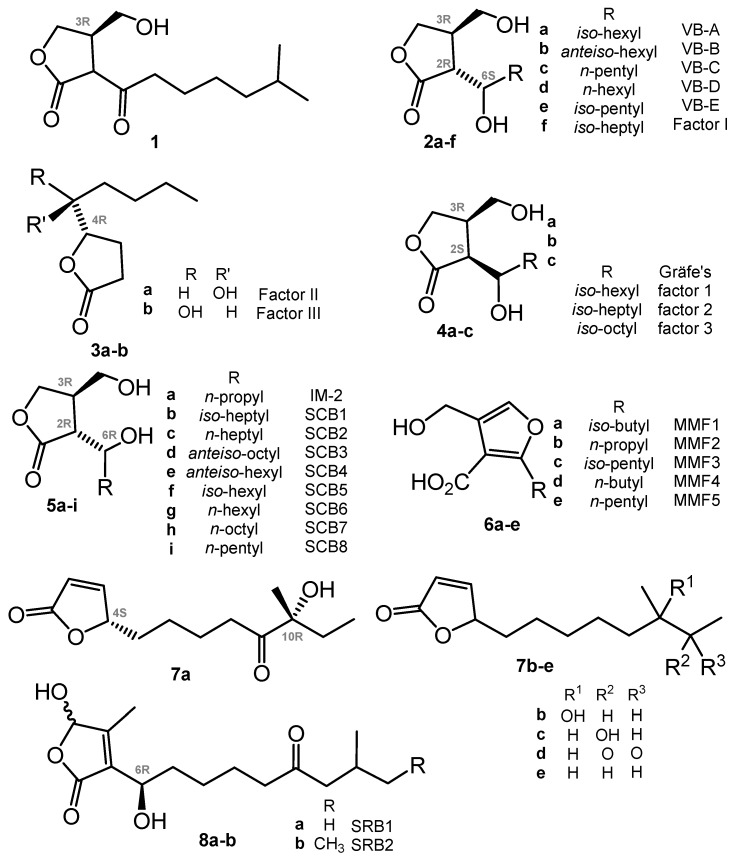
GBL and closely related regulators: butenolides (avenolide, *S. rochei* butenolides or SRB) and furans (methylenomycin furans or MMF).

Historically, A-factor (**1**, Chart 1) (A for “autoregulation”) was the first compound that revolutionized our views of the secondary metabolism and development cycle of actinomycetes. This γ-butyrolactone (GBL) derivative was discovered by Prof. Khokhlov and co-workers in 1967 [22].

A-factor acts as a pleiotropic regulator: it binds to the A-factor receptor protein (ArpA) and causes dissociation of this suppressor from DNA. This triggers the transcription of the *adpA* gene encoding the transcription activator AdpA, which in turn induces morphological differentiation, spore formation, and biosynthesis of secondary metabolites [23,24].

In further decades, many other closely related autoregulators were discovered, e.g., *Streptomyces virginiae* butanolides (VBs A-E, **2a**–**e**) [25,26,27], Factor I (**2f**) [28], Factors II and III (**3a**,**b**) [29], Gräfe’s factors (**4a**–**c**) from *S. bikiniensis* and *S. cyaneofuscatus* [30], IM-2 (**5a**) [31,32], SCB1 (**5b**) [33,34], SCB2,3 (**5c**,**d**) [35] and SCB4–8 (**5e**–**i**) [36], methylenomycin furans (MMFs, **6**) [37], avenolide (**7a**) from *S. avermitilis* [38], related compounds (**7b**–**e**) from *S. albus* [39], and two *S. rochei* butenolides (SRBs, **8a**,**b**) [40]. The stereoconfiguration of avenolide analogues **7b**–**e** reported very recently has not been assigned [39]. All these compounds have a high degree of similarity in their chemical nature (butanolides, butenolides, or furans), biosynthesis pathways, and in mechanisms of action on actinomycetes. The transduction of the chemical signal starts from binding with a specific receptor protein (like ArpA in the A-factor signaling cascade) belonging to the TetR family of transcriptional regulators; the binding of compounds probably causes conformational changes, thus inducing the relocation of the DNA-binding domain and DNA release [23].

TetR transcriptional repressors are well represented and widespread in bacteria [41,42]. The recent study on the distribution of the GBL (IM-2, VB)-type and butenolide (avenolide)-type signaling showed these hormones to be common in actinomycetes. Production of regulators was screened using *S. lavendulae* FRI-5 (Δ*farX*), *S. virginiae* (Δ*barX*), and *S. avermitilis* (Δ*aco*) strains with disrupted essential biosynthetic genes for the corresponding hormone-like molecules. Twenty percent of the investigated strains produced **5a** (IM-2), another 20% produced VBs, and 24% of actinomycetes showed avenolide activity [43].

Thus, the potential for various applications of A-factor-like regulators is quite high: they induce biosynthesis of antibiotics of different classes, e.g., aminoglycosides (streptomycin), streptogramins (virginamycin), peptide ionophores (valinomycin) and others, including clinically significant antibiotics. For more detail see ref. [24].

## 3. Other Types of (auto)Regulators

**Chart 2:**
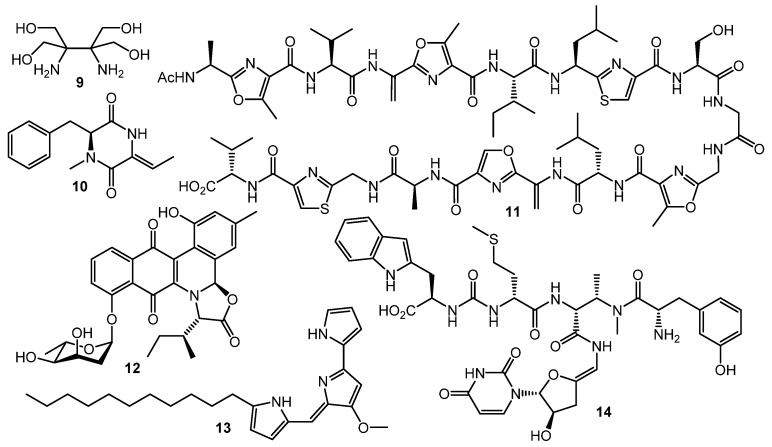
Other (non-GBL) types of regulators.

However, GBLs and related compounds are not the only type of regulators found in actinomycetes. Hitherto, a few different types of autoregulators were reported (Chart 2), e.g., the pi-factor from *S. natalensis* (**9**) [44] and l-*N*-methylphenylalanyl-dehydrobutyrine diketopiperazine (**10**) from *S. globisporus* [45]. These molecules have no structural similarity to GBL-type compounds and their mechanisms of action remain unclear [46,47].

Goadsporin (**11**) [48,49], an active 19-aa peptide found in the culture broth of *Streptomyces* sp. TP-A0584, shows extremely broad elicitation activity on actinobacteria. Induction of sporulation and growth inhibition were observed in about 80% of *Streptomyces* sp. and *Micromonospora* sp. isolates after goadsporin treatment [50]. Possible mechanism of the goadsporin action on susceptible cells was postulated by Prof. H. Onaka and co-workers. Bioinformatics analysis of *godI*—self-resistance gene from the goadsporin BGC—showed a high degree of homology with *ffh* encoding the Ffh protein. The Ffh and 4.5S RNA form the signal recognition particles (SRPs) in prokaryotic cells. The SPRs recognize the signal peptide of membrane or secretory proteins and facilitate their translocation to the cytoplasmic membrane. Binding of Ffh protein with goadsporin causes the upregulation of protein translocation and maturation, growth inhibition, and alternation of secondary metabolism [51].

It was suggested that goadsporin and its analogs [51,52] could be useful in crop protection: *S. scabies*, which is known to cause potato scab, is effectively inhibited by the low concentrations of goadsporin.

Two “atypical” transcriptional regulators were discovered in *S. venezuelae*: JadR1 activates the transcription of jadomycin B (**12**) biosynthetic genes while repressing its own gene. Jadomycin B binds to the N-terminal receiver domain of JadR1, causing dissociation of JadR1 from target promoters. High expression of the biosynthetic enzyme genes leads to accumulation of the product in a concentration sufficient to inhibit the initial activation. Therefore, jadomycin acts as an autoregulator of its own synthesis. Similarly, the RedZ protein regulating production of undecylprodigiosine (**13**) is modulated by the end product. These findings demonstrate that end-product-mediated control of antibiotic biosynthesis may be widespread in nature, offering seminal basis for interspecies signaling and interaction [53,54]. Production of another class of antibiotics, uridyl peptides sansanmycins (sansanmycin A, **14**), active against multidrug-resistant *Mycobacterium tuberculosis*, is also controlled at the transcriptional level by the end-product-mediated mechanism [55].

**Chart 3:**
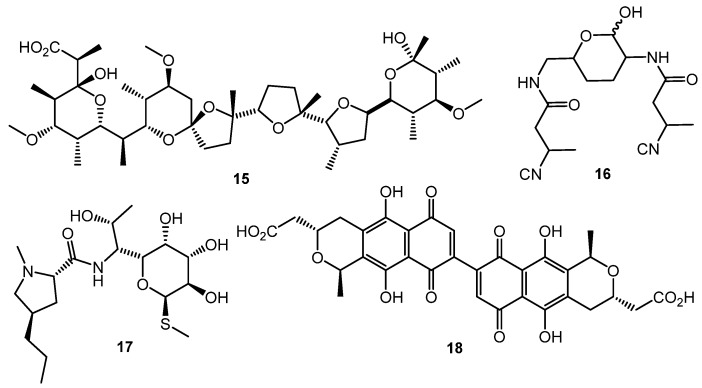
Antibiotic-antibiotic induction; elicitors and products.

Polyether antibiotics at sub-inhibitory concentrations can elicit the synthesis of secondary metabolites. Promomycin (**15**, Chart 3) and closely related compounds (salinomycin, monensin, and nigericin) promoted the synthesis of cryptic isonitrile antibiotic SF2768 (**16**) by *S. griseorubiginosus* [56,57]. This was the first example of isolation of a novel original compound using the chemical elicitation approach. However, the action of polyether antibiotics has not yet been studied on the molecular level.

Lincomycin (**17**) at one tenth of its MIC increased the expression of the pathway-specific regulatory gene actII-ORF4 in the enzyme gene cluster producing the blue-pigmented antibiotic actinorhodin (**18**) in *S. lividans*, thus resulting in actinorhodin overproduction [58]. Chloramphenicol activated biosynthetic genes at the transcriptional level and increased the amino acid pool 1.5- to 6-fold, enhancing the production of non-ribosomal peptide antibiotics [59]. Other ribosome-targeted antibiotics, especially thiosteptone and spectinomycin, at sub-inhibitory concentrations were also capable of altering colony morphology and antibiotics (**13** and **18**) production in *S. coelicolor* M145 [60].

**Chart 4:**
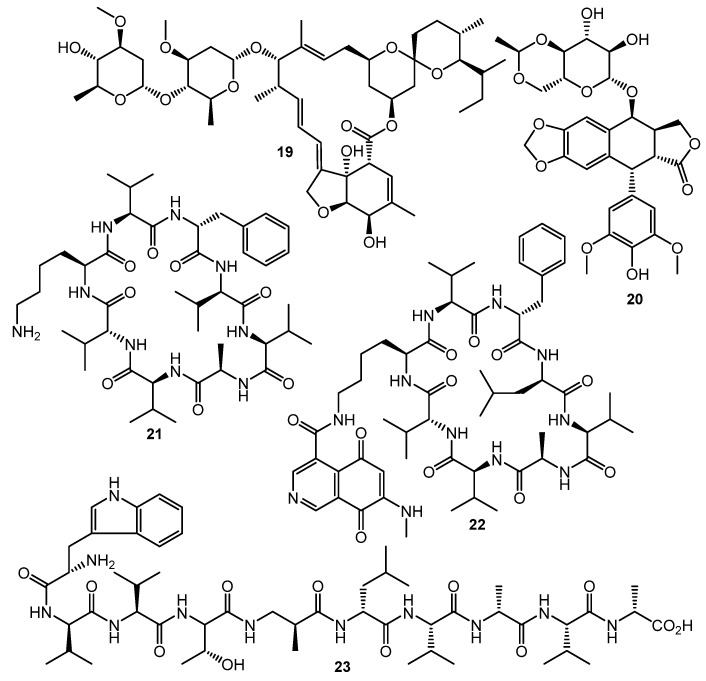
Natural products eliciting surugamide biosynthesis in *S. albus* J1074 (detected by HiTES).

The HiTES (High-Throughput Elicitor Screening) approach [61,62,63] was developed to discover small molecule elicitors of silent biosynthetic gene clusters and novel secondary metabolites using a simple fluorescent assay format. Three copies of *eGFP* (enhanced green fluorescent protein encoding gene) inserted in the *S. albus* J1074 genome both at a neutral site (*attB*) and inside the biosynthetic cluster of interest (*sur*, surugamide BGC). The difference in fluorescence between *attB::Psur-eGFPx3* (the *Psur* promoter region (~260 bp) upstream of *surE*) and *surE::eGFPx3* strains after testing compound treatment was used as an indicator of BGC’s activation. Screening of a commercially-available natural products library (ca. 500 compounds) identified 6 potential elicitors, among these ivermectin b1a (**19**, Chart 4) and etoposide (**20**) had the maximum surugamide BGC activation effect and markedly changed the secondary metabolome profile of *S. albus* J1074—14 novel small molecules were isolated and characterized using these two inducers, e.g., surugamide J (**21**), albucyclone F (**22**), and surugamide F2 (**23**) [63].

## 4. Metabolism Remodeling and Epigenetic Control

**Chart 5:**
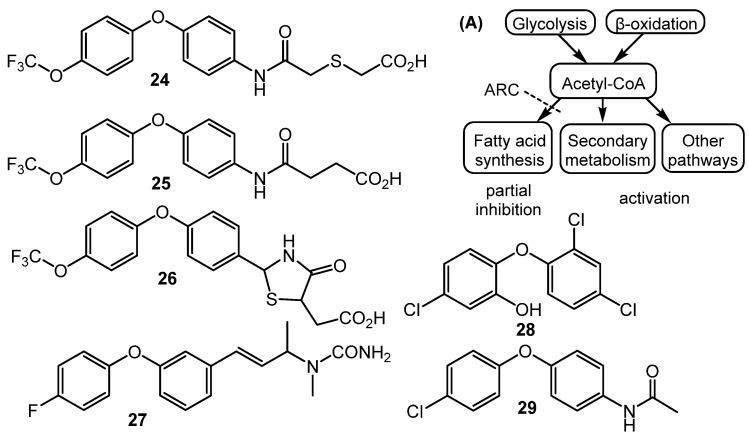
Synthetic diphenyl ethers (ARCs) as elicitors and their mode of action (**A**).

**Chart 6:**
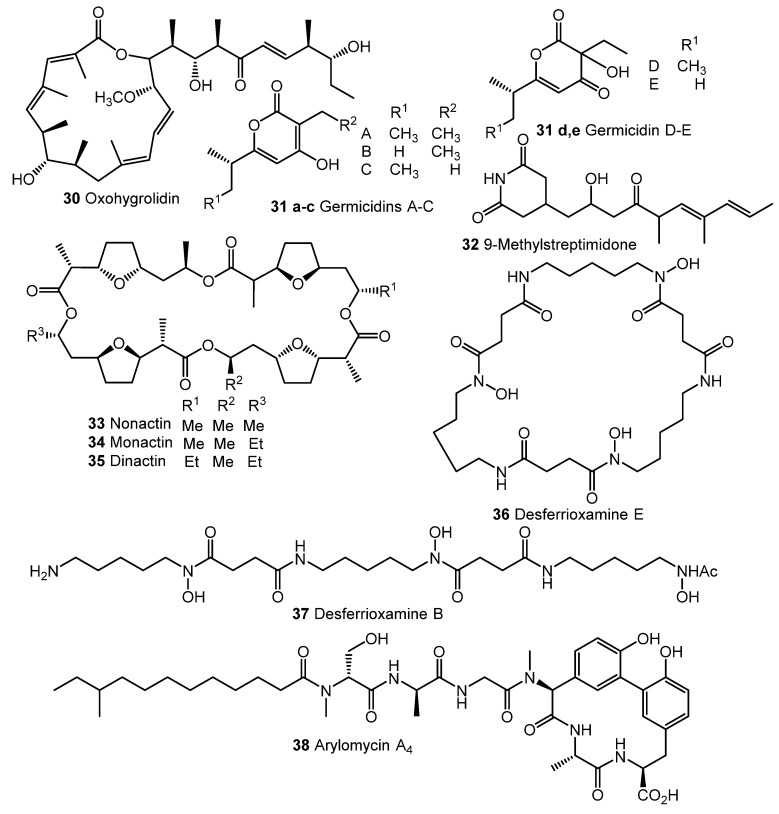
Cl-ARC-induced biosynthesis: examples of identified antibiotics [67].

Fully synthetic chemicals also can be utilized as elicitors. ARC2 (**24**, ARC from “Antibiotic-Remodeling-Compound”) and similar diphenyl ethers (ARC3–5, **25**–**27**) (Chart 5) have become the first inducer examples discovered by systematic screening of 30,569 small molecules [64,65]. Taking into account the structural similarity of ARC compounds with triclosan (**28**), a well-known biocide and inhibitor of fatty acid biosynthesis, the mechanism of ARCs’ action (Chart 5A) has been proposed. Generally, ARCs act as metabolism remodeling compounds, changing the balance between primary and secondary metabolic pathways. The mode of action was confirmed by the experimental data: ARCs changed fatty acid pool by inhibition of key biosynthetic enzymes like FabI. Remarkably, triclosan also can stimulate polyketide antibiotic synthesis at sub-inhibitory concentrations [59]. Further investigation of the different ARCs’ effects on the streptomycetes biochemistry revealed the targets for different molecules (even structurally close) as not being the same; however, all of them influenced fatty acid metabolism [66]. Chlorinated analog Cl-ARC (**29**) was used to enhance the expression of cryptic biosynthetic genes, and the observed difference between growth on control and elicitor-containing media was studied using a metabolomics approach. More than 100 induced secondary metabolites were detected, including rare antibiotics [67]. Several identified structures are represented in Chart 6: oxohygrolidin (**30**), germicidins A–E (**31a**–**e**) 9-methylstreptimidone (**32**), nactins (**33**, **34**, **35**), desferrioxamines B,E (**36**, **37**), and arylomycin A_4_ (**38**).

**Chart 7:**
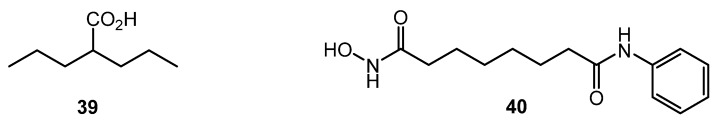
Histone deacetylase (HDAC) inhibitors.

Despite the significant difference in structural DNA organization between eukaryotes and actinomycetes, inhibitors of histone deacetylase (HDAC) like valproic acid (**39**) and suberanilohydroxamic acid (SAHA, **40**) (Chart 7) showed effect on antibiotic production by several *Streptomyces* strains, especially on nutrient-poor media. The finding was explained by the homology between several *S. coelicolor* enzymes (SCO0452, SCO6464, SCO3330) and human HDAC enzymes [68]. Epigenetic modification could be used for the control of antibiotic biosynthesis; the approach, however, is not well studied for actinobacteria.

## 5. Conclusions

Activation of antibiotics’ biosynthesis in actinomycetes by using small-molecule elicitors seems to be a useful technique for drug discovery. The approach allows identification of new metabolite scaffolds and enhances synthesis of low yield secondary metabolites. Moreover, antibiotic-antibiotic induction relationships are believed to be the basis of chemical signaling between different actinobacterial strains and are, therefore, of interest for chemical ecology. Elicitation is an obvious cost-effective approach in biotechnology for increasing antibiotic yields and reducing fermentation time.

## References

[1] WHO Publishes List of Bacteria for Which New Antibiotics Are Urgently Needed. http://www.who.int/mediacentre/news/releases/2017/bacteria-antibiotics-needed/en/.

[2] The National Strategy to Prevent the Antimicrobial Resistance Spreading (2017–2030). http://government.ru/docs/29477/.

[3] Nett M., Ikeda H., Moore B.S. (2009). Genomic basis for natural product biosynthetic diversity in the actinomycetes. Nat. Prod. Rep..

[4] Rutledge P.J., Challis G.L. (2015). Discovery of microbial natural products by activation of silent biosynthetic gene clusters. Nat. Rev. Microbiol..

[5] Genilloud O. (2017). Actinomycetes: Still a source of novel antibiotics. Nat. Prod. Rep..

[6] Yamanaka K., Reynolds K.A., Kersten R.D., Ryan K.S., Gonzalez D.J., Nizet V., Dorrestein P.C., Moore B.S. (2014). Direct cloning and refactoring of a silent lipopeptide biosynthetic gene cluster yields the antibiotic taromycin A. Proc. Natl. Acad. Sci. USA.

[7] Nah H.-J., Pyeon H.-R., Kang S.-H., Choi S.-S., Kim E.-S. (2017). Cloning and heterologous expression of a large-sized natural product biosynthetic gene cluster in *Streptomyces* species. Front. Microbiol..

[8] Alberti F., Khairudin K., Venegas E.R., Davies J.A., Hayes P.M., Willis C.L., Bailey A.M., Foster G.D. (2017). Heterologous expression reveals the biosynthesis of the antibiotic pleuromutilin and generates bioactive semi-synthetic derivatives. Nat. Commun..

[9] Wei Y., Zhang L., Zhou Z., Yan X. (2018). Diversity of gene clusters for polyketide and nonribosomal peptide biosynthesis revealed by metagenomics analysis of the Yellow Sea sediment. Front. Microbiol..

[10] Suroto D.A., Kitani S., Arai M., Ikeda H., Nihira T. (2018). Characterization of the biosynthetic gene cluster for cryptic phthoxazolin A in *Streptomyces avermitilis*. PLoS ONE.

[11] Bode H.B., Bethe B., Höfs R., Zeeck A. (2002). Big effects from small changes: Possible ways to explore nature’s chemical diversity. ChemBioChem.

[12] Reen F.J., Romano S., Dobson A.D.W., O’Gara F. (2015). The sound of silence: Activating silent biosynthetic gene clusters in marine microorganisms. Mar. Drugs.

[13] Liu M., Grkovic T., Liu X., Han J., Zhang L., Quinn R.J. (2017). A systems approach using OSMAC, Log P and NMR fingerprinting: An approach to novelty. Synth. Syst. Biotechnol..

[14] Abdelmohsen U.R., Grkovic T., Balasubramanian S., Kamel M.S., Quinn R.J., Hentschel U. (2015). Elicitation of secondary metabolism in actinomycetes. Biotechnol. Adv..

[15] Van der Meij A., Worsley S.F., Hutchings M.I., van Wezel G.P. (2017). Chemical ecology of antibiotic production by actinomycetes. FEMS Microbiol. Rev..

[16] Wang G., Hosaka T., Ochi K. (2008). Dramatic activation of antibiotic production in *Streptomyces coelicolor* by cumulative drug resistance mutations. Appl. Environ. Microbiol..

[17] Tojo S., Tanaka Y., Ochi K. (2015). Activation of antibiotic production in *Bacillus* spp. by cumulative drug resistance mutations. Antimicrob. Agents Chemother..

[18] Ochi K. (2017). Insights into microbial cryptic gene activation and strain improvement: Principle, application and technical aspects. J. Antibiot..

[19] Zhu H., Sandiford S.K., van Wezel G.P. (2014). Triggers and cues that activate antibiotic production by actinomycetes. J. Ind. Microbiol. Biotechnol..

[20] Okada B.K., Seyedsayamdost M.R. (2017). Antibiotic dialogues: Induction of silent biosynthetic gene clusters by exogenous small molecules. FEMS Microbiol. Rev..

[21] Zarins-Tutt J.S., Barberi T.T., Gao H., Mearns-Spragg A., Zhang L., Newman D.J., Goss R.J.M. (2016). Prospecting for new bacterial metabolites: A glossary of approaches for inducing, activating and upregulating the biosynthesis of bacterial cryptic or silent natural products. Nat. Prod. Rep..

[22] Khokhlov A.S., Tovarova I.I., Borisova L.N., Pliner S.A., Shevchenko L.N., Kornitskaia E.I., Ivkina N.S., Rapoport I.A. (1967). The A-factor, responsible for streptomycin biosynthesis by mutant strains of *Actinomyces streptomycini*. Dokl. Akad. Nauk SSSR.

[23] Niu G., Chater K.F., Tian Y., Zhang J., Tan H. (2016). Specialised metabolites regulating antibiotic biosynthesis in *Streptomyces* spp.. FEMS Microbiol. Rev..

[24] Efremenkova O.V. (2016). A-factor-like autoregulators. Russ. J. Bioorg. Chem..

[25] Yanagimoto M., Terui G. (1971). Physiological studies on staphylomycin production II, formation of a substance effective in inducing staphylomycin production. J. Ferment. Technol..

[26] Yanagimoto M., Yamada Y., Terui G. (1979). Extraction and purification of inducing material produced in staphylomycin fermentation. Hakko Kogaku Kaishi.

[27] Yamada Y., Sugamura K., Kondo K., Yanagimoto M., Okada H. (1987). The structure of inducing factors for virginiamycin production in *Streptomyces virginiae*. J. Antibiot..

[28] Gräfe U., Schade W., Eritt I., Fleck W.F., Radics L. (1982). A new inducer of anthracycline biosynthesis from *Streptomyces viridochromogenes*. J. Antibiot..

[29] Gräfe U., Reinhardt G., Schade W., Krebs D., Eritt I., Fleck W.F., Heinrich E. (1982). Isolation and structure of novel autoregulators from *Streptomyces griseus*. J. Antibiot..

[30] Gräfe U., Reinhardt G., Schade W., Eritt I., Fleck W.F., Radics L. (1983). Interspecific inducers of cytodifferentiation and anthracycline biosynthesis from *Streptomyces bikinensis* and *S. cyaneofuscatus*. Biotechnol. Lett..

[31] Sato K., Nihira T., Sakuda S., Yanagimoto M., Yamada Y. (1989). Isolation and structure of a new butyrolactone autoregulator from *Streptomyces* sp. FRI-5. J. Ferment. Bioeng..

[32] Hashimoto K., Nihira T., Sakuda S., Yamada Y. (1992). IM-2, a butyrolactone autoregulator, induces production of several nucleoside antibiotics in *Streptomyces* sp. FRI-5. J. Ferment. Bioeng..

[33] Takano E., Nihira T., Hara Y., Jones J.J., Gershater C.J.L., Yamada Y., Bibb M. (2000). Purification and structural determination of SCB1, a γ-butyrolactone that elicits antibiotic production in *Streptomyces coelicolor* A3(2). J. Biol. Chem..

[34] Takano E., Chakraburtty R., Nihira T., Yamada Y., Bibb M. (2001). A complex role for the γ-butyrolactone SCB1 in regulating antibiotic production in *Streptomyces coelicolor* A3(2). Mol. Microbiol..

[35] Hsiao N.-H., Nakayama S., Merlo M.E., de Vries M., Bunet R., Kitani S., Nihira T., Takano E. (2009). Analysis of two additional signaling molecules in *Streptomyces coelicolor* and the development of a butyrolactone-specific reporter system. Chem. Biol..

[36] Sidda J.D., Poon V., Song L., Wang W., Yang K., Corre C. (2016). Overproduction and identification of butyrolactones SCB1–8 in the antibiotic production superhost *Streptomyces* M1152. Org. Biomol. Chem..

[37] Corre C., Song L., O’Rourke S., Chater K.F., Challis G.L. (2008). 2-Alkyl-4-hydroxymethylfuran-3-carboxylic acids, antibiotic production inducers discovered by *Streptomyces coelicolor* genome mining. Proc. Natl. Acad. Sci. USA.

[38] Kitani S., Miyamoto K.T., Takamatsu S., Herawati E., Iguchi H., Nishitomi K., Uchida M., Nagamitsu T., Omura S., Ikeda H. (2011). Avenolide, a *Streptomyces* hormone controlling antibiotic production in *Streptomyces avermitilis*. Proc. Natl. Acad. Sci. USA.

[39] Nguyen T.B., Kitani S., Shimma S., Nihira T. (2018). Butenolides from *Streptomyces albus* J1074 act as external signals to stimulate avermectin production in *Streptomyces avermitilis*. Appl. Environ. Microbiol..

[40] Arakawa K., Tsuda N., Taniguchi A., Kinashi H. (2012). The butenolide signaling molecules SRB1 and SRB2 induce lankacidin and lankamycin production in *Streptomyces rochei*. ChemBioChem.

[41] Ramos J.L., Martínez-Bueno M., Molina-Henares A.J., Terán W., Watanabe K., Zhang X., Gallegos M.T., Brennan R., Tobes R. (2005). The TetR family of transcriptional repressors. Microbiol. Mol. Biol. Rev..

[42] Cuthbertson L., Nodwell J.R. (2013). The TetR family of regulators. Microbiol. Mol. Biol. Rev..

[43] Thao N.B., Kitani S., Nitta H., Tomioka T., Nihira T. (2017). Discovering potential *Streptomyces* hormone producers by using disruptants of essential biosynthetic genes as indicator strains. J. Antibiot..

[44] Recio E., Colinas A., Rumbero A., Aparicio J.F., Martin J.F. (2004). PI factor, a novel type quorum-sensing inducer elicits pimaricin production in *Streptomyces natalensis*. J. Biol. Chem..

[45] Matselyukh B., Mohammadipanah F., Laatsch H., Rohr J., Efremenkova O., Khilya V. (2015). *N*-methylphenylalanyl-dehydrobutyrine diketopiperazine, an A-factor mimic that restores antibiotic biosynthesis and morphogenesis in *Streptomyces globisporus* 1912-B2 and *Streptomyces griseus* 1439. J. Antibiot..

[46] Recio E. (2006). Glycerol, ethylene glycol and propanediol elicit pimaricin biosynthesis in the PI-factor-defective strain *Streptomyces natalensis npi287* and increase polyene production in several wild-type actinomycetes. Microbiology.

[47] Vicente C.M., Santos-Aberturas J., Guerra S.M., Payero T.D., Martín J.F., Aparicio J.F. (2009). PimT, an amino acid exporter controls polyene production via secretion of the quorum sensing pimaricin-inducer PI-factor in *Streptomyces natalensis*. Microb. Cell Fact..

[48] Onaka H., Tabata H., Igarashi Y., Sato Y., Furumai T. (2001). Goadsporin, a chemical substance which promotes secondary metabolism and morphogenesis in *streptomycetes*. I. Purification and characterization. J. Antibiot..

[49] Igarashi Y., Kan Y., Fujii K., Fujita T., Harada K.-I., Naoki H., Tabata H., Onaka H., Furumai T. (2001). Goadsporin, a chemical substance which promotes secondary metabolism and morphogenesis in *streptomycetes*. II. Structure determination. J. Antibiot..

[50] Onaka H. (2017). Novel antibiotic screening methods to awaken silent or cryptic secondary metabolic pathways in actinomycetes. J. Antibiot..

[51] Onaka H., Nakaho M., Hayashi K., Igarashi Y., Furumai T. (2005). Cloning and characterization of the goadsporin biosynthetic gene cluster from *Streptomyces* sp. TP-A0584. Microbiology.

[52] Ozaki T., Yamashita K., Goto Y., Shimomura M., Hayashi S., Asamizu S., Sugai Y., Ikeda H., Suga H., Onaka H. (2017). Dissection of goadsporin biosynthesis by in vitro reconstitution leading to designer analogues expressed in vivo. Nat. Commun..

[53] Wang L., Tian X., Wang J., Yang H., Fan K., Xu G., Yang K., Tan H. (2009). Autoregulation of antibiotic biosynthesis by binding of the end product to an atypical response regulator. Proc. Natl. Acad. Sci. USA.

[54] Wang W., Ji J., Li X., Wang J., Li S., Pan G., Fan K., Yang K. (2014). Angucyclines as signals modulate the behaviors of *Streptomyces coelicolor*. Proc. Natl. Acad. Sci. USA.

[55] Li Q., Wang L., Xie Y., Wang S., Chen R., Hong B. (2013). SsaA, a member of a novel class of transcriptional regulators, controls sansanmycin production in *Streptomyces* sp. strain SS through a feedback mechanism. J. Bacteriol..

[56] Amano S., Morota T., Kano Y., Narita H., Hashidzume T., Yamamoto S., Mizutani K., Sakuda S., Furihata K., Takano-Shiratori H. (2010). Promomycin, a polyether promoting antibiotic production in *Streptomyces* spp.. J. Antibiot..

[57] Amano S., Sakurai T., Endo K., Takano H., Beppu T., Furihata K., Sakuda S., Ueda K. (2011). A cryptic antibiotic triggered by monensin. J. Antibiot..

[58] Imai Y., Sato S., Tanaka Y., Ochi K., Hosaka T. (2015). Lincomycin at subinhibitory concentrations potentiates secondary metabolite production by *Streptomyces* spp.. Appl. Environ. Microbiol..

[59] Tanaka Y., Izawa M., Hiraga Y., Misaki Y., Watanabe T., Ochi K. (2017). Metabolic perturbation to enhance polyketide and nonribosomal peptide antibiotic production using triclosan and ribosome-targeting drugs. Appl. Microbiol. Biotechnol..

[60] Wang H., Zhao G., Ding X. (2017). Morphology engineering of *Streptomyces coelicolor* M145 by sub-inhibitory concentrations of antibiotics. Sci. Rep..

[61] Seyedsayamdost M.R. (2014). High-throughput platform for the discovery of elicitors of silent bacterial gene clusters. Proc. Natl. Acad. Sci. USA.

[62] Okada B.K., Wu Y., Mao D., Bushin L.B., Seyedsayamdost M.R. (2016). Mapping the trimethoprim-induced secondary metabolome of *Burkholderia thailandensis*. ACS Chem. Biol..

[63] Xu F., Nazari B., Moon K., Bushin L.B., Seyedsayamdost M.R. (2017). Discovery of a cryptic antifungal compound from *Streptomyces albus* J1074 using high-throughput elicitor screens. J. Am. Chem. Soc..

[64] Craney A., Ozimok C., Pimentel-Elardo S.M., Capretta A., Nodwell J.R. (2012). Chemical perturbation of secondary metabolism demonstrates important links to primary metabolism. Chem. Biol..

[65] Ochi K., Okamoto S. (2012). A magic bullet for antibiotic discovery. Chem. Biol..

[66] Ahmed S., Craney A., Pimentel-Elardo S.M., Nodwell J.R. (2013). A synthetic, species-specific activator of secondary metabolism and sporulation in *Streptomyces coelicolor*. ChemBioChem.

[67] Pimentel-Elardo S.M., Sørensen D., Ho L., Ziko M., Bueler S.A., Lu S., Tao J., Moser A., Lee R., Agard D. (2015). Activity-independent discovery of secondary metabolites using chemical elicitation and cheminformatic inference. ACS Chem. Biol..

[68] Moore J.M., Bradshaw E., Seipke R.F., Hutchings M.I., McArthur M. (2012). Use and discovery of chemical elicitors that stimulate biosynthetic gene clusters in *Streptomyces* bacteria. Meth. Enzymol..

